# Benefits of Regional Anesthesia Versus Opioids in the Management of Postoperative Pain Following Excision of a Peripheral Nerve Sheath Tumor

**DOI:** 10.14740/jmc5369

**Published:** 2026-07-28

**Authors:** Duveen Nalamothu, Mauricio Arce Villalobos, Joseph D. Tobias

**Affiliations:** a Department of Internal Medicine, Doctors Hospital and OhioHealth, Columbus, OH, USA; b Department of Anesthesiology and Pain Medicine, Nationwide Children’s Hospital, Columbus, OH 43205, USA; c Department of Anesthesiology and Pain Medicine, The Ohio State University College of Medicine, Columbus, OH, USA; d Department of Pediatrics, The Ohio State University College of Medicine, Columbus, OH, USA

**Keywords:** Continuous nerve sheath catheter, Peripheral nerve analgesia, Postoperative pain management, Opioids, Regional anesthesia

## Abstract

Postoperative opioid exposure in opioid-naive patients may carry a risk of persistent opioid use. We present the case of a 16-year-old female with no significant family history who was recently diagnosed with neurofibromatosis type 1 and found to have a rapidly enlarging mass in her left upper extremity. She was scheduled for excisional biopsy. To minimize the need for postoperative opioids, analgesia via a continuous peripheral nerve catheter was employed following resection of the malignant peripheral nerve sheath tumor. This case report reviews the clinical applications of multimodal analgesia via a continuous peripheral nerve catheter in pediatric patients, including placement techniques and dosing considerations.

## Introduction

Peripheral nerve sheath tumors present a surgical challenge, as their anatomy predisposes patients to significant postoperative neuropathic and nociceptive pain [1]. Conventional pain management strategies often rely on parenteral opioids; however, these approaches raise important concerns, including the risk of persistent postoperative opioid use, physical dependence, and addiction [2–4]. These factors, combined with the ongoing opioid crisis, which was estimated to be responsible for approximately 73,000 deaths in 2025, have intensified interest in minimizing postoperative opioid exposure through multimodal analgesic strategies [5].

Continuous peripheral nerve analgesia, such as that administered via a peripheral nerve catheter (PNC), may provide superior postoperative analgesia compared to intravenous opioids across a variety of surgical procedures [6]. This benefit is particularly evident in upper and lower extremity orthopedic surgeries, where the analgesic effect can be titrated and maintained over several days. When incorporated into comprehensive multimodal analgesia protocols, continuous peripheral nerve analgesia may allow for complete avoidance of opioids in the postoperative period [7]. We report use of a PNC in a 16-year-old female following excisional biopsy of a malignant peripheral nerve sheath tumor. The use of PNC for postoperative analgesia is described, with emphasis on placement techniques and dosing regimens.

## Case Report

This retrospective review was approved by the Institutional Review Board of Nationwide Children’s Hospital (Columbus, OH, USA). The study was conducted in accordance with the hospital’s ethical standards for research involving human subjects and in compliance with the principles of the Declaration of Helsinki.

A 16-year-old, 47.7-kg adolescent with a prior diagnosis of neurofibromatosis (NF) presented with the insidious onset of pain in her left upper extremity, followed by the development of a palpable mass in the same region. Over the course of 8 months, the mass progressively enlarged, leading to associated numbness and decreased motor function in the left hand. Magnetic resonance imaging of the upper extremity revealed a large, lobulated solid mass with central fluid signal characteristics, concerning for a malignant peripheral nerve sheath tumor in the setting of underlying NF. The lesion demonstrated partial encasement of the brachial artery and vein, as well as periosteal involvement of the humeral diaphysis. A whole-body positron emission tomography scan showed a fluorodeoxyglucose (FDG)-avid mass in the left upper arm with central necrosis. Two FDG-avid left axillary lymph nodes were also identified, representing either reactive changes or regional lymph node involvement. Computed tomography of the chest demonstrated enlarged axillary lymph nodes without evidence of pulmonary nodules.

The patient was scheduled for excisional biopsy. A postoperative pain management plan was developed utilizing a continuous PNC, specifically, an interscalene catheter, with a low-concentration local anesthetic infusion for postoperative pain control. The goal was to provide effective analgesia while preserving motor function to avoid interference with the surgical team’s postoperative neurologic assessment.

**Figure 1:**
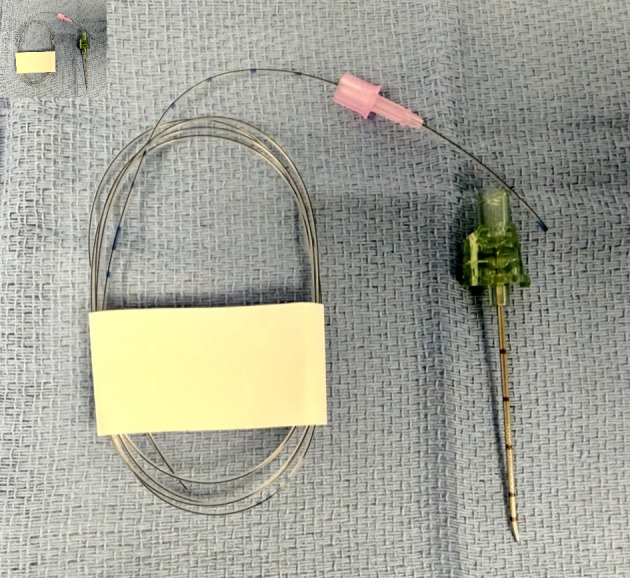
A peripheral nerve catheter from a B. Braun Contiplex^®^ Tuohy Ultra 360 set was placed under ultrasound guidance between the superior and middle trunks of the brachial plexus using an interscalene approach. The catheter was placed through a 5-cm, 18-gauge Tuohy regional blockade needle.

**Figure 2:**
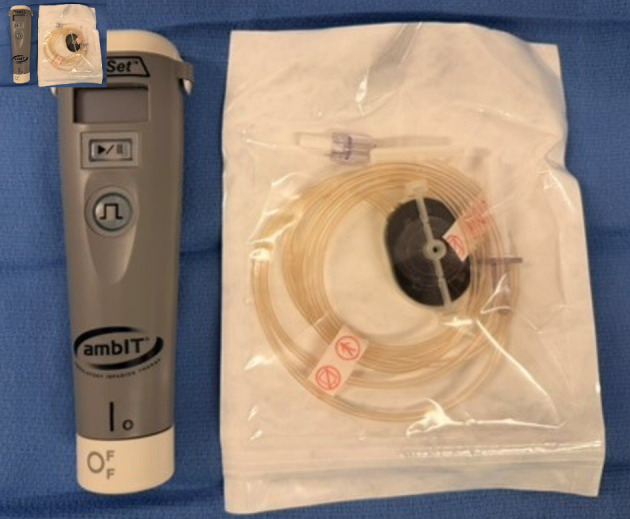
Ropivacaine (0.1%) was infused through the peripheral nerve catheter at home using the Avanos^®^ AmbIT pain control system. The catheter was subsequently removed at home without complications on postoperative day 5.

On the day of surgery, the patient was transported to the preoperative area. She reported no history of adverse reactions to previous anesthetic procedures and denied any family history of anesthesia-related complications. Vital signs were within normal limits. The preoperative evaluation was unremarkable except for the presence of an upper extremity mass. The patient also reported paresthesia of the left thumb, first, and second digits, along with decreased grip strength. She was classified as American Society of Anesthesiologists (ASA) Physical Status III. Special considerations included the risk of blood loss and potential nerve injury, as the tumor was located in close proximity to the neurovascular bundle of the affected extremity. The patient was kept *nil per os* for 8 h prior to the procedure, and no premedication was administered. She was transported to the operating room, where standard ASA monitors were applied. A 20-gauge peripheral intravenous cannula was inserted on the dorsal aspect of the right hand. Anesthesia was induced intravenously with midazolam (2 mg), fentanyl (100 µg), and propofol (4 mg/kg). Bag-valve-mask ventilation was easy, and the airway was secured with a size 3 laryngeal mask airway (LMA). General anesthesia was maintained with sevoflurane at an end-tidal concentration of 1.8–2.2% in a mixture of air and oxygen. Cefazolin was administered for prophylaxis against a postoperative surgical site infection. Following the induction of anesthesia, a left-sided interscalene PNC was placed under sterile conditions. The superior, middle, and inferior trunks of the left brachial plexus (interscalene approach) were identified under ultrasound and a 5-cm, 18-gauge Tuohy regional blockade needle (B. Braun Contiplex^®^ Tuohy Ultra 360 Set, Fig. 1) was advanced under ultrasound guidance until the tip was positioned between the superior and middle trunks. Hydro-dissection with 0.9% sodium chloride was performed to facilitate placement, and a 20-gauge catheter was advanced through the needle with its tip located between these structures. The catheter was secured to the skin using a Tegaderm™ CHG dressing. At this time, the catheter was not dosed. The surgical procedure lasted approximately 120 min. Intraoperative findings revealed a large mass with a necrotic center arising from the ulnar nerve, with associated extrinsic compression of the brachial artery and vein. Due to the extent of involvement, complete resection of the mass without injury to the ulnar nerve was not feasible. The ulnar nerve was therefore transected, after which an excisional biopsy was successfully performed. Neurorrhaphy was not possible following excision, as the distance between the proximal and distal ends of the ulnar nerve measured approximately 10.5 cm. Estimated blood loss during the procedure was 50 mL. Additional intraoperative medications included dexamethasone (4 mg), ondansetron (4 mg), and hydromorphone (0.5 mg). No neuromuscular blocking agents were administered during the procedure, in accordance with prior discussion with the surgical team. Emergence from anesthesia was uneventful. Once the patient demonstrated adequate spontaneous ventilation, protective airway reflexes, and appropriate tidal volumes, the LMA was removed, and she was transferred to the post-anesthesia care unit (PACU) in stable condition. Following completion of the physical examination and documentation of neurological deficits related to the surgical procedure, PNC dosing was initiated with a bolus dose of 0.1% ropivacaine (0.1 mL/kg), followed by a continuous infusion at 4 mL/h. Pain scores in PACU were less than 3 (self-report numerical rating scale of 1–10). The patient was discharged from the PACU 30 min after arrival. Her postoperative course was reassuring, with pain scores of 0/10 and no requirement for opioid analgesia. The analgesic regimen consisted of a continuous infusion of 0.1% ropivacaine at 4 mL/h via the PNC, in addition to oral acetaminophen (650 mg every 6 h). Neurological examination demonstrated the expected deficits in the ulnar nerve distribution; however, motor and sensory function of the radial and median nerves remained intact. Beginning on postoperative day 1, the patient successfully participated in physical therapy and continued to progress appropriately. Given the adequate trajectory of her postoperative course and effective pain management with the ability to fully participate in physical therapy, the decision was made to continue the same infusion regimen via the PNC. The patient was discharged home on postoperative day 3. Analgesic infusion via the PNC was continued at home using the Avanos^®^ AmbIT pain control system (Fig. 2). The catheter was subsequently removed at home without complications on postoperative day 5.

## Discussion

As discussed in this case report, our patient was able to have an early discharge from the hospital with her PNC in place. In order to achieve this successfully, there are key elements that need to be present. Appropriate patient selection includes reliable and motivated patients and/or caregivers, who are capable of following home instructions. Contraindications to catheter placement include coagulation disturbances, true local anesthetic allergy, or infection at the insertion site. Comprehensive preoperative education should be provided through both verbal and written instructions covering pump operation, catheter management, recognition of complications, and proper catheter removal techniques. Postoperative follow-up should provide patients and caregivers with continuous 24/7 telephone access to an anesthesiologist or acute pain service, supplemented by structured daily follow-up calls to monitor analgesia and identify potential complications. PNC removal may be safely performed at home by patients or caregivers under telephone guidance, although removal can also be completed during a postoperative surgical follow-up visit. With these concerns in mind, a multimodal analgesic regimen including a PNC in combination with supplemental oral medications (acetaminophen, NSAIDs, and rescue opioids as needed), can be used to provide superior analgesia and facilitate earlier hospital discharge.

In our patient, the interscalene approach was chosen given our familiarity with this approach to the brachial plexus and the perceived lower incidence of adverse effects (pneumothorax) when compared to the supraclavicular approach. The interscalene approach to the brachial plexus is known to provide excellent analgesia for the shoulder and proximal humerus; however, coverage of the ulnar nerve territory, which originates predominantly from the lower trunk, may be less reliable. A supraclavicular approach may provide more consistent blockade of the ulnar nerve distribution and could be considered more suitable for upper arm surgery. With the advance of ultrasound-guided techniques, the supraclavicular approach has been shown to be safe and effective and has therefore increased in our clinical practice. Additionally, in our patient, the extent of the sensory block was determined primarily by the provision of excellent analgesia. Given that transection of the ulnar nerve was necessary during the surgical procedure, our patient may be at greater risk of chronic neuropathic pain in the ulnar distribution, and a more selective approach to the lower trunk may theoretically have been beneficial in prevention of long-term consequences of nerve transection.

Motor deficits resulting either from true nerve injury from the surgical procedure or the residual effects of local anesthetic agents are potential adverse effects related to peripheral regional anesthetic techniques. In a previous study of benign peripheral nerve sheath tumors, new postoperative motor deficits were observed in approximately 6.3% of cases [8]. Similarly, studies involving limb schwannomas have reported that the postoperative neurologic deficit rate is 12.7% [9]. Preservation of motor function is therefore a critical component of the postoperative physical examination performed by the surgical team. For this reason, regional anesthesia is sometimes avoided from a surgical standpoint in favor of systemic opioids, despite their associated adverse effects.

However, distinguishing true nerve injury from the effects of regional anesthesia using local anesthetic agents relies primarily on the temporal course and clinical pattern of recovery. Local anesthetic agents produce a distribution-specific motor and sensory deficit that corresponds to the expected duration of the agent used. This typically ranges from approximately 1–3 h with lidocaine to 6–12 h with longer-acting agents such as bupivacaine or ropivacaine. Resolution in these cases is characteristically progressive and complete. In contrast, true nerve injury presents as a conduction deficit that may persist for weeks or longer, depending on the severity of injury. When a motor deficit persists beyond 24–48 h, a residual local anesthetic effect becomes increasingly unlikely [10]. In the present case, the patient demonstrated residual neurologic deficits in the distribution of the ulnar nerve, which were attributable to the surgical procedure itself. These were identified prior to dosing of the PNC for postoperative analgesia.

Moreover, the rationale for using local anesthetics is based on the differential nerve blockade principle. The nervous system detects nociceptive signals primarily using small diameter, thinly myelinated A-delta (Aδ) fibers and unmyelinated C-fibers. Whereas motor function is mediated by large-diameter, heavily myelinated A-alpha (Aα) fibers. Local anesthetic agents, which generally have a high pKa, predominately exist in ionized forms at physiological pH allowing them to penetrate the thin Aδ and C-fibers. However, the myelin sheath of the Aα fibers act as a diffusion barrier thus limiting motor blockade [11]. Dilution of the local anesthetic agent such as 0.1% ropivacaine used in our patient, is generally able to provide sensory nerve blockade while limiting motor blockade [12]. Therefore, leveraging this principle, a low-concentration local anesthetic infusion was employed to optimize analgesia while preserving motor function, enabling participation in physical therapy and maintaining adequate pain control to support early hospital discharge. This was chosen based on our usual clinical practice when postoperative neurologic evaluation is required to identify neurologic sequelae or deficits from the surgical procedure. In general, 0.1% ropivacaine is able to provide effective analgesia while limiting the impact on motor function and allowing ongoing neurologic assessment of the patient [13]. If analgesia had remained inadequate, a higher concentration of the local anesthetic solution (0.15–0.2%) can be considered.

Compared with opioids, regional anesthesia has demonstrated superior outcomes in postoperative pain management following various surgical procedures. Techniques involving regional or local anesthesia were identified as key factors associated with improved postoperative pain scores at 2, 12, and 48 h in a meta-analysis encompassing more than 800 studies across more than 50 countries [14]. Without regional anesthesia, neither opioid-inclusive nor opioid-free strategies have consistently demonstrated superior analgesic efficacy in this context. Additionally, prolonged PACU stays have been observed in cases where regional anesthetic techniques were not utilized. The use of regional anesthesia has also been shown to reduce postoperative opioid requirements by 50%, as well as the incidence of chronic pain and persistent opioid use [15].

Although regional anesthesia has been shown to be an effective means of providing postoperative analgesia, single shot techniques will provide only 8–12 h of analgesia. As major orthopedic procedures may result in acute postoperative pain lasting 2–3 days, more prolonged methods of providing postoperative analgesia are needed. Hospital and home PNC programs have been shown to be safe and effective following various surgical procedures initially in adults and subsequently in pediatric-aged patients [16–22]. Continuous peripheral nerve blocks (CPNBs) have been established as a cornerstone of multimodal postoperative analgesia in adults with benefits ranging from decreased baseline pain, improved sleep, more efficient discharge readiness, decreased opioid use, and limited opioid-related adverse effects. Similar benefits have been noted in pediatric-aged patients where studies have shown that the majority of patients may not require the limited use of supplemental opioids. A longitudinal study of 1,285 children discharged home with CPNBs demonstrated that 75.4% required no supplemental opioids or only as-needed oral opioids, with a mean catheter duration of approximately 51 h [19, 23]. No neurologic deficits were observed at 6-month follow-up. Subsequent pediatric studies have confirmed the feasibility, analgesic efficacy, and high satisfaction associated with this approach. Although awake placement may be feasible in adults, when used in pediatric-aged patients, PNCs are generally placed under general anesthesia.

Bupivacaine and ropivacaine are the two most commonly utilized local anesthetic agents for analgesia via PNCs in pediatric patients. Infusion rates are typically limited to 0.2–0.3 mg/kg/h in children. Dosing is further limited to 0.1–0.2 mg/kg/h in neonates and infants because of the heightened risk of local anesthetic systemic toxicity (LAST) related to immature hepatic metabolism and reduced protein binding. Chloroprocaine has also been used as an alternative agent, with recommended infusion rates not exceeding 12 mg/kg/h, although systemic toxicity is uncommon given its rapid ester hydrolysis and favorable pharmacokinetic characteristics [24].

Consistent with literature, major complications associated with this analgesic modality are uncommon in both adult and pediatric populations. While catheter colonization rates have been reported between 6–46%, clinically significant infections remain rare, with reported rates of 0.1–2.9%. Established risk factors include catheter duration exceeding 48 h, intensive care unit (ICU) admission, lack of antibiotic prophylaxis, and femoral, axillary, or groin catheter locations. Therefore, ASRA Pain Medicine guidelines strongly emphasize adherence to strict aseptic technique during catheter placement [25]. Clinically relevant neurologic injury related to PNCs has been shown to be uncommon in both adult and pediatric patients. Catheter dislodgement, obstruction, migration, and leakage may occur; however, these events are typically minor and rare. LAST is exceedingly uncommon when dilute, low-concentration local anesthetic solutions are administered within recommended dose limits [7, 26, 27].

### Learning points

Current guidelines recommend incorporating regional anesthesia as part of a multimodal analgesia strategy to improve analgesia, shorten the postoperative inpatient course, and limit postoperative opioid use. The physiologic effects of regional anesthesia including PNCs may extend beyond effective analgesia, as ongoing clinical evidence has linked regional anesthesia with additional beneficial physiologic effects. Blocking afferent nociceptive signals with regional anesthesia may blunt the neuroendocrine surgical stress response, reduce the release of catecholamines and cortisol, attenuate systemic inflammation and decrease the incidence of delirium, hyperalgesia, and chronic pain development [28].

Ideally, opioid medications should be reserved as rescue adjuncts rather than the primary analgesic technique. Multimodal pain management has undergone substantial developments in recent years and continues to expand with the use of home PNCs and local anesthetic infusions using deposable devices. This approach is particularly important in the context of the ongoing opioid epidemic, in which both pediatric and adult populations remain at risk for opioid dependence and persistent postoperative opioid use. The current study demonstrates the feasibility of home use of a PNC to control pain following surgical excision of a large peripheral nerve sheath tumor. Multidisciplinary cooperation between the perioperative anesthesia and surgical teams allowed the controlled use of a dilute local anesthetic infusion while permitting ongoing neurologic examination in a patient with the potential for postoperative neurologic deficits.

## Data Availability

Any inquiries regarding supporting data availability of this study should be directed to the corresponding author.

## References

[1] Raasveld FV, Hanna T, Pacheco FJ, Gonzalez MR, Johnston B, Lozano-Calderon SA, Valerio IL (2025). Neuropathic pain following surgery for malignant peripheral nerve sheath tumors. Ann Surg Oncol.

[2] Harbaugh CM, Lee JS, Hu HM, McCabe SE, Voepel-Lewis T, Englesbe MJ, Brummett CM (2018). Persistent opioid use among pediatric patients after surgery. Pediatrics.

[3] Cina RA, Ward RC, Basco WT, Taber DJ, Gebregziabher M, McCauley JL, Lockett MA (2022). Incidence and patterns of persistent opioid use in children following appendectomy. J Pediatr Surg.

[4] Langnas E, Lin A, Luo Y, Rodriguez-Monguio R, Bicket M, Cairo S, De Vaan M (2025). The association of opioid prescriptions to children after discharge from surgery and new opioid prescription fills in family members. J Pediatr.

[5] Dowell D, Nataraj N, Rikard M, Park J, Zhang K, Baldwin G (2025). Why have overdose deaths decreased? Widespread fentanyl saturation and decreased drug use among key drivers. Lancet Reg Health Am.

[6] Cardwell TW, Zabala V, Mineo J, Ochner CN (2022). The effects of perioperative peripheral nerve blocks on peri- and postoperative opioid use and pain management. Am Surg.

[7] Joshi G, Gandhi K, Shah N, Gadsden J, Corman SL (2016). Peripheral nerve blocks in the management of postoperative pain: challenges and opportunities. J Clin Anesth.

[8] Desai KI (2017). The surgical management of symptomatic benign peripheral nerve sheath tumors of the neck and extremities: an experience of 442 cases. Neurosurgery.

[9] Raj C, Amouyel T, Maynou C, Chantelot C, Saab M (2024). Limb schwannoma: Factors for postoperative neurologic deficit and poor functional results. Orthop Traumatol Surg Res.

[10] Brull R, Hadzic A, Reina MA, Barrington MJ (2015). Pathophysiology and etiology of nerve injury following peripheral nerve blockade. Reg Anesth Pain Med.

[11] Wildsmith JA (2025). Axonal sensitivity and block dynamics. Reg Anesth Pain Med.

[12] Christiansen CB, Madsen MH, Molleskov E, Rothe C, Lundstrom LH, Lange KHW (2020). The effect of ropivacaine concentration on common peroneal nerve block duration using a fixed dose: A randomised, double-blind trial in healthy volunteers. Eur J Anaesthesiol.

[13] Fettiplace M, Joudeh L, Bungart B, Boretsky K (2024). Local anesthetic dosing and toxicity of pediatric truncal catheters: a narrative review of published practice. Reg Anesth Pain Med.

[14] de Carvalho CC, El-Boghdadly K, Guedes IHL, Dantas MVM, Tome DPB, Ramos IB, Gomes CSH (2026). Opioid-free vs. opioid-inclusive anaesthesia with or without regional anaesthesia for postoperative pain: a systematic review with network meta-analysis of randomised controlled trials. Anaesthesia.

[15] Pepper CG, Mikhaeil JS, Khan JS (2024). Perioperative regional anesthesia on persistent opioid use and chronic pain after noncardiac surgery: a systematic review and meta-analysis of randomized controlled trials. Anesth Analg.

[16] Ilfeld BM (2011). Continuous peripheral nerve blocks: a review of the published evidence. Anesth Analg.

[17] Ganesh A, Rose JB, Wells L, Ganley T, Gurnaney H, Maxwell LG, DiMaggio T (2007). Continuous peripheral nerve blockade for inpatient and outpatient postoperative analgesia in children. Anesth Analg.

[18] Ilfeld BM, Smith DW, Enneking FK (2004). Continuous regional analgesia following ambulatory pediatric orthopedic surgery. Am J Orthop (Belle Mead NJ).

[19] Gurnaney H, Kraemer FW, Maxwell L, Muhly WT, Schleelein L, Ganesh A (2014). Ambulatory continuous peripheral nerve blocks in children and adolescents: a longitudinal 8-year single center study. Anesth Analg.

[20] Visoiu M, Joy LN, Grudziak JS, Chelly JE (2014). The effectiveness of ambulatory continuous peripheral nerve blocks for postoperative pain management in children and adolescents. Paediatr Anaesth.

[21] Bhalla T, Dairo OO, Martin DP (2014). A proactive risk assessment by utilizing healthcare failure mode and effect analysis (HFMEA) for safe implementation of peripheral nerve catheters in pediatric patients. Anaesth Pain Intensive Care.

[22] Gable A, Burrier C, Stevens J, Wrona S, Klingele K, Bhalla T, Martin DP (2016). Home peripheral nerve catheters: the first 24 months of experience at a children's hospital. J Pain Res.

[23] Simic D, Stevic M, Stankovic Z, Simic I, Ducic S, Petrov I, Milenovic M (2018). The safety and efficacy of the continuous peripheral nerve block in postoperative analgesia of pediatric patients. Front Med (Lausanne).

[24] Veneziano G, Tobias JD (2017). Chloroprocaine for epidural anesthesia in infants and children. Paediatr Anaesth.

[25] Provenzano DA, Hanes M, Hunt C, Benzon HT, Grider JS, Cawcutt K, Doshi TL (2025). ASRA Pain Medicine consensus practice infection control guidelines for regional anesthesia and pain medicine. Reg Anesth Pain Med.

[26] Dadure C, Capdevila X (2012). Peripheral catheter techniques. Paediatr Anaesth.

[27] Swenson JD, Bay N, Loose E, Bankhead B, Davis J, Beals TC, Bryan NA (2006). Outpatient management of continuous peripheral nerve catheters placed using ultrasound guidance: an experience in 620 patients. Anesth Analg.

[28] Reysner T, Wieczorowska-Tobis K, Kowalski G, Grochowicka M, Pyszczorska M, Mularski A, Reysner M (2024). The influence of regional anesthesia on the systemic stress response. Reports (MDPI).

